# Recovery of Prenatal Baicalein Exposure Perturbed Reproduction by Postnatal Exposure of Testosterone in Male Mice

**DOI:** 10.1155/2020/5012736

**Published:** 2020-11-25

**Authors:** Sridevi Vaadala, Naveen Ponneri, Venkata Shashank Karanam, Sri Bhashyam Sainath, Pamanji Sreenivasuala Reddy, Ramachandra Reddy Pamuru, Arifullah Mohammed

**Affiliations:** ^1^Department of Biochemistry, Yogi Vemana University, Vemanapuram, Kadapa 516 005, AP, India; ^2^West High School, Torrance 90503, CA, USA; ^3^Department of Biotechnology, Vikrama Simhapuri University, Kakutur, P. S. Nellore 524 320, AP, India; ^4^Department of Zoology, Sri Venkateswara University, Tirupati 517 502, AP, India; ^5^Institute of Food Security and Sustainable Agriculture (IFSSA), Universiti Malaysia Kelantan Campus Jeli, Locked Bag 100, Jeli 17600, Kelantan, Malaysia; ^6^Faculty of Agro-based Industry (FIAT), Universiti Malaysia Kelantan Campus Jeli, Locked Bag 100, Jeli 17600, Kelantan, Malaysia

## Abstract

Baicalein (BC), a flavonoid, which lacks the qualities of reproductive health and shows adverse effects, is tested in this study. Inseminated mice were injected with 30, 60, and 90 mg BC/Kg body weight on gestation days 11, 13, 15, and 17. The F1 BC-exposed males of each dosage were divided into six groups. First three groups (*n* = 6 from each BC dosage) were used for assessment of reproductive performance, the others (*n* = 4 from each BC dosage) were administered with testosterone 4.16 mg/kg body weight on postnatal days 21, 31, and 41. The reproductive health of adult F1 males at the age of 55 and 60 was tested. Prenatal BC exposure showed reduced fertility after cohabitation with control females. The BC exposure significantly reduced the body weight, tissue indices, and sperm parameters (motility, count, viability, and daily sperm count) and altered the sperm membrane in a hypoosmotic swelling test. A downward trend was observed in testicular steroidogenic marker enzymes (3*β*- and 17*β*-steroid dehydrogenases) and serum testosterone, whereas increase in serum titers of FSH and LH along with altered the testicular histology. Conversely, testosterone (4.16 mg/kg body weight) partially recovered reduced male reproductive health by BC. BC impaired male reproductive health due to low levels of testosterone is reverted by external testosterone is evidenced in this study.

## 1. Introduction

The development of male reproductive tract is very sensitive to changes in hormones including estrogens, and thus minute changes can adversely affect the reproductive functions. In male, the correlation between reproduction and estrogen signalling occurs via the expression of estrogen receptors in the testis and accessory sex organs at all developmental stages: fetal, neonatal, and adult periods [1]. Earlier, it has been shown that the phytoestrogen-induced developmental and reproductive toxicity occurs via estrogen signalling [2, 3]. Previously, the reproductive toxic effects of phytoestrogen genistein, diadzein, and coumestrol have been demonstrated [4]. Studies of Brooks and Thompson [5] also indicated that the phytoestrogens can interfere with the steroidogenic pathway and subsequently affect androgen synthesis. A reduction in the testosterone levels has also been reported in coumestrol-exposed rats [6]. Neonatal studies indicated that the exposure of rats to estrogens deteriorates the testosterone levels and Sertoli cell number, and causes rete tubule distension associated with reduced fertility efficacy [7, 8]. Furthermore, there are evidences towards the adverse effects of prenatal exposure to phytoestrogens on male reproduction such as deterioration of testosterone production, sperm motility, and fertility efficacy in animal models, raising concerns about the future fertility [9, 10]. It has also been shown that the exposure of male infants to phytoestrogens did not affect the male reproduction in animal models [4]. Thus, it is apparent that the data available so far on phytoestrogens and the male reproduction have been conflicting. Moreover, as most of the data are majorly obtained from the animal models exposed to genistein and diadzein, studies addressing other phytoestrogenic compounds might be helpful to get a clear-cut picture on probable effects of phytoestrogens on male reproductive health.

Baicalein (BC: 5, 6, 7 trihydroxyflavone) is a phytoestrogen that belongs to flavonoid family and is obtained from the mint plant family member, *Scutellaria baicalensis* (Chinese skullcap). It is commonly used as a component of Sho-Saiko-To to treat chronic hepatitis [11]. It acts as anti-inflammatory [12], antithrombotic, antidepressant, anticancer, and neuroprotective agents [13, 14]. BC is also widely applied to reduce blood cholesterol levels [15], and it also acts as an inhibitor of the alternate complement pathway [16]. Recently, the antiviral properties of baicalein, a metabolite of baicalein against dengue virus, have been reported [17]. With regard to male reproduction, studies of Jiang et al. [18] indicated that the baicalein can regulate the expression of connexin43 (proteins that sustain the intercellular communication between the Sertoli cells) in TM4 Sertoli cells of mice testes. Most notably, the ingredients of *Scutellaria radix* including baicalein have been detected in the early and late embryonic tissues of pregnant rats using HPLC/MS analysis [19], suggesting that the baicalein is able to cross placental barrier. However, data related to the effect of prenatal exposure to baicalein on male reproduction are poorly understood.

Considering the facts from the above literature, it can be submitted that (1) several studies related to phytoestrogens in animal models focused on the testosterone levels, (2) prenatal exposure to phytoestrogens such as genistein and diadzein adversely affects male reproductive health, (3) studies on the effects of prenatal exposure to baicalein on testicular functions are not well defined, and (4) because of the therapeutic potential, flavonoids including baicalein are widely consumed by humans. The purpose of this study was twofold: firstly, we addressed which reproductive endpoints were affected in F1 mice exposed to baicalein during prenatal period, and secondly, we evaluated whether the supplementation of testosterone reverses the affected reproductive endpoints in F1 mice exposed to baicalein during gestation.

## 2. Materials and Methods

### 2.1. Procurement of Mice and Husbandry

The test animal, adult female albino mice weighing 29 ± 2 g (45–50 days old) were purchased from Sri Venkateswara Enterprises (Bangalore, India). Animals were housed in a clean, well-ventilated, and air-conditioned room (12 h: 12 h light: dark cycle) at 25 ± 2°C with a relative humidity of 50 ± 5% at the animal house facility, Yogi Vemana University, Kadapa, A.P., India. Sterilized polypropylene cages lined with paddy husk were used to house the mice and supplemented sterilized feed (purchased from Sri Venkateswara Enterprises, Bangalore, Karnataka, India) and water *ad libitum*. A week after, adaptive mice were used for trialling. Experiments were performed in concurrence with the guidelines of the Committee for the Purpose of Control and Supervision on Experiments on Animals (Government of India) (CPCSEA) by following the rules for care and use of laboratory animals (NRC, 1996)adopted by the Institutional (Yogi Vemana University) Animal Ethical Committee (resolution no: 1841/GO/Re/S/15/CPCSEA dt. 18-11-2015) [20].

### 2.2. Test Chemicals

Commercially available baicalein (BC) was purchased (Sigma Aldrich, USA) and dissolved in 100% dimethyl sulfoxide (DMSO). Readily available testosterone depot (100 mg/ml, German remedies, Goa, India) was procured (a local medical store) and used for recovery studies.

### 2.3. Design of Testing

Inseminated female mice (24 numbers) identified through incidence of sperm in the vaginal smear cytology and/or vaginal plug were equally divided into 4 groups. The day female inseminated was considered as gestation day (GD) 1 and confirmed the pregnancy with increased body weight and size of mice until 7^th^ day after insemination and maintained them in individual cages. Randomly selected pregnant mice were divided into control which received 100% DMSO or testing groups administered with BC 30, 60, and 90 mg/kg body weight (dissolved in 100% DMSO) on GD 11, 13, 15, and 17. The dosage of BC selected in the present study was similar to the dosage given for rats and mice in the previous studies (30–100 mg/kg body weight for intravenous and intraperitoneal) [21, 22]. The pregnant mice were allowed to deliver pups, and at day 21, F1 generation males were separated. The prenatal BC-exposed males of each dosage was divided into two subgroups. The first subgroup (*n* = 6 from each BC dosage) was used for assessment of reproductive performance, and the other subgroup (*n* = 4 from each BC dosage) was administered with testosterone 4.16 mg/kg body weight/mice [23] on postnatal days (PND) 21, 31, and 41. Respective control groups were also maintained.

### 2.4. F1 Generation Fertility Study

On PND 55, BC-exposed male mice injected with or without testosterone were individually assessed for fertility. The reproductive fecundity of F1 males were assessed by observing the reproductive end points such as conception time, mating index, fertility index, resorptions/mice, number of live fetuses, and postimplantations loss. F1 males were cohabitated with virgin females (1 male: 1 female) at proestrus stage of estrus cycle [24], and the cohabitation period was six days. Everyday morning from the day of cohabitation, vaginal smear was analyzed for the presence of sperm. The presence of sperm in vaginal smear was considered as day 1 of pregnancy and inseminated females were maintained in separate cages. At GD 18, the pregnant mice were autopsied and the uterus was analyzed for postimplantation loss, and live and dead fetuses.

### 2.5. Necropsy

After completion of fertility studies, control and experimental groups were analyzed for selected reproductive endpoints such as sperm parameters, testicular steroidogenic enzyme activities (3*β*- and 17*β*-HSD), and testicular histology. Prior necropsy, the body weights were recorded, and the animals were humanely sacrificed by cervical dislocation. Blood was withdrawn immediately after necropsy through cardiac puncture and allowed to settle down for the separation of serum and stored at −80°C until further analysis of the hormones such as testosterone (T), follicle stimulating hormone (FSH), and luteinizing hormone (LH).

#### 2.5.1. F1 Male Body Weight Organ/Tissue Collection and it's Somatic Indices

The brain, Liver, kidney, and reproductive organs were immediately isolated, cleared from adhesive tissues and blood, and weighed to their nearest milligram. The tissue somatic indices were calculated using the following formula:

(1) $$\begin{array}{c} \text{tissue somatic index }\left(\text{TSI}\right)=\left[\frac{\text{weight of the organ }\left(\text{g}\right)}{\text{weight of the animal }\left(\text{g}\right)}\right]\times 100\text{.} \end{array}$$

#### 2.5.2. F1 Generation Sperm Analysis

The testis was analyzed for daily sperm count, and cauda epididymis was used to analyze the sperm parameters such as sperm count, sperm motility, sperm viability, and number of tail coiled sperm (the hypoosmotic swelling test: the HOS-test). Animals from control and experimental groups were individually analyzed for selected sperm endpoints. Immediately after squeezing the cauda epididymis in 0.9% NaCl (physiological saline), sperm motility was analyzed at 37°C in 5 min [25] and evaluated sperm count (millions mL−1) using the Neubauer chamber in 15 min under a phase contrast microscope (Olympus, BX 43 Japan). Using trypan blue (1%) reagent, sperm viability was evaluated [26]. The tail coiling of sperms was recorded under the phase contrast microscope using an HOS tail coiling method described earlier by Jeyendran et al. [27] where the hypoosmotic solution causes an influx into the sperm membrane. This resulted in coiling of the tail, whereas abnormal sperm did not respond to the HOS-test.

The number of sperm produced/g tissue/day was considered as daily sperm production (DSP) and was measured by following the method described by Blazak et al. [28]. In short, decapsulated testis was grinded in 50 mL of 0.01% Triton X-100 containing ice-cold saline (0.9% NaCl) and each sample was thoroughly mixed after allowing the lysate to settle down for 1 min followed by counting the homogenization resistant sperm using a haemocytometer.

#### 2.5.3. Testicular Steroidogenic Enzyme Assay in F1 Males

The testicular titers of 3*β*-HSD (E.C. 1.1.1.51) and 17*β*-HSD (E.C. 1.1.1.61) in control and experimental mice were determined by following the method of Bergmeyer [29]. Briefly, the testis (10% W/V) homogenized in ice-cold 20 mM Tris-HCl buffer (pH 6.8) was subjected to centrifugation steps to obtain the microsomal portion. This was carefully aspirated into a fresh tube and used as enzyme source. About 2.0 ml of final reaction mixture consists of sodium pyrophosphate buffer (pH 9.0) 100 *μ*moles, NAD (cofactor) 0.5 µmoles, and substrate (dehydroepiandrosterone) 0.08 µmoles, and enzyme source (equivalent of 20 mg microsomal protein) for 3*β*-HSD. Substrate (0.08 *μ*moles androstenedione) and cofactors (0.5 *μ*moles NADPH) are replaced for 17*β*-HSD. With 20-second intervals, absorbance was measured at 340 nm for 5 minutes using a UV-Vis spectrophotometer (LABINDIA, UV-3092). The quantity of protein present in enzyme fraction was estimated by following the method of Lowry et al. [30] where bovine serum albumin was used as a standard. The *n* moles of NAD converted to NADH/mg protein/min (3*β*-HSD) and the *n* moles of NADPH converted to NADP/mg protein/min (17*β*-HSD) were calculated to express the testicular steroidogenic enzyme activity.

#### 2.5.4. F1 Male Serum Hormone Analysis

With the sensitivity of 0.002 ng, the serum testosterone titer was estimated using the method of Rao et al. [31] by radioimmunoassay and 6.5% of intra-assay variation was found. In the serum of control and experimental mice, FSH and LH titers were estimated according to Lin et al. [32]. According to the method described by Greenwood et al. [33], rFSH and rLH iodination was done in presence of chloramines-T (oxidizing agent) with ^125^I. The level of sensitivity for FSH and LH found in this test is 0.008 ng and 0.006 ng, respectively. An intra-assay variations 5 (FSH) and 6 (LH) percent found were avoided by running all samples at a time.

#### 2.5.5. F1 Male Testicular Histological Study

The testis fixed in formalin (10%) was transferred to Bouin's fluid and incubated for 24 hrs. Desiccated Bouin's fixed specimens were treated with an ascending graded series of alcohol and were entrenched in paraffin wax. Sections were incised at a thickness of 5 *μ*m and were stained with hematoxylin-eosin after deparaffinization with xylene [34]. Histological sections were observed under a microscope (BX 43, Olympus, Japan) and photographed.

### 2.6. Statistical Analysis

The two-tailed ANOVA followed by the Bonferroni post-test to compare replicate means by row was used to analyze data statistically by using GraphPad.Prism.v5.0.3.477. The results were expressed as mean ± SEM, and *P* < 0.05 was considered as statistically significant.

## 3. Results

### 3.1. Toxicity

Behaviour, body position, coordination or gait activeness, and overall appearance of experimental mice were observed and recorded time to time. No mice were excluded from the study, and no clinical sign of toxicity was recorded during the experimentation. No difference was recorded between control and experimental mice in respiration, urination, water and food intake, lacrimation, vocalization, and postural positions. The behavioural postures such as head flicking, biting, circling, head searching, walking backwards, and self-mutilation were absolutely comparable to control and experimental mice.

### 3.2. F1 Generation Fertility

**Figure 1:**
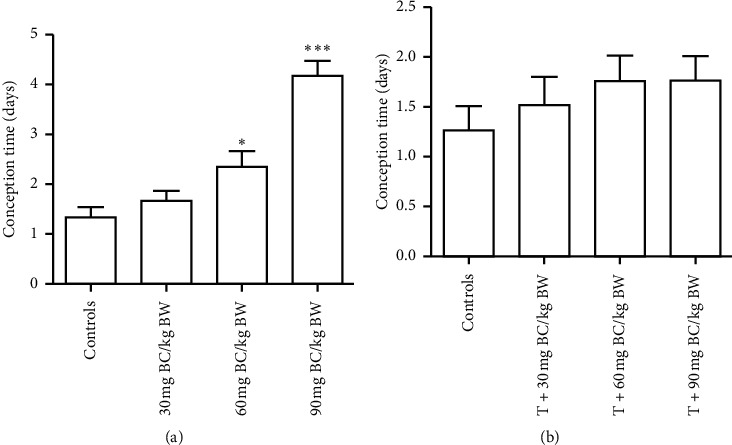
Conception time of females cohabitated with both baicalein (BC)-exposed (a) without (*n* = 6) or (b) with (*n* = 4) testosterone (T)-injected male mice; ^*∗*^ and ^*∗∗∗*^ represent significant difference from controls at *P* < 0.05 and <0.001, respectively, where BW = body weight.

**Figure 2:**
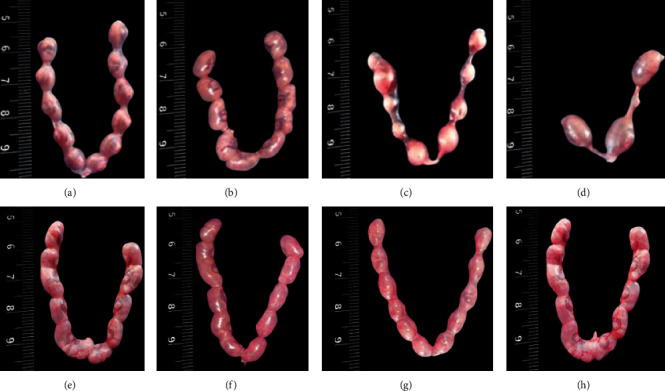
Postimplantations on the 18^th^ day of pregnancy of female mice cohabitated both baicalein (BC)-exposed without or with testosterone (T)-injected male mice. (a) and (e) Control. (b), (c), and (d) 30, 60, and 90 mg·BC/kg body weight (BW) exposed F1 males, respectively; (f), (g), and (h) 30, 60, and 90 mg·BC/kg BW exposed F1 males administered with 4.16 mg·T/kg BW, respectively.

The F1 males (exposed to BC and BC with testosterone) were evaluated for fertility efficacy and selected reproductive endpoints. A significant decrease (*P* < 0.05) in the number of corpora lutea, implantations, and live fetuses associated with a significant increase in the number of resorptions was observed in mice cohabited with BC-exposed males and a significant increase (*P* < 0.05) in the conception time and the number of resorptions was recorded in BC-exposed F1 males (Table 1). The decrease in percentage of fertility index, pre-and postimplantation loss, implantations, resorptions, and resorption index was found in F1 BC-exposed males. All the control females cohabitated with control males showed 100% copulatory index and fertility index. The control females mated with F1 BC-exposed males were showing 100% mating index (occurrence of copulatory plugs), but fertility index decreased with the increased dosage of BC (83.3%, 66.6%, and 50% for 30, 60, and 90 mg BC/kg BW, respectively), whereas in BC with testosterone-treated mice, 100% mating index along with increased fertility index (100%, 75%, and 75% for 30, 60, and 90 mg BC/kg BW with testosterone, respectively) was shown. The mean conception time was 1.33 days in the controls, and it was increased to 1.66, 2.33, and 4.16 days in F1 BC-exposed male mice vs control females (Figure 1(a)) compared to control males (1.25 days) and control females vs F1 BC with testosterone-treaded males (1.5, 1.75, and 1.75 days, respectively for 30, 60, and 90 mg BC/kg BW exposed with testosterone) (Figure 1(b)). The number of corpora lutea per mice significantly decreased (*P* < 0.05) in females mated with F1 BC-exposed males in contrast to control females vs control males and control females vs F1 BC with testosterone-exposed males. Significant decrease (*P* < 0.0001) in the mean number of implantations and live fetuses per mice was found in F1 males exposed to BC. In contrast, the mean number of implantations (10.25 ± 0.29, 10.25 ± 0.25, and 10.25 ± 0.47) and live fetuses per mice (9.50 ± 0.29, 9.75 ± 0.25, and 10 ± 0.41) was found comparable among control females mated with F1 males exposed to 30, 60, and 90 mg BC/kg BW with testosterone. Percentage of implantations decreased in F1 males exposed to 30, 60, and 90 mg BC/kg BW (95.94, 91.26, and 86.24%, respectively). No significant change in resorption percentage was observed in the control females mated with control males and control females vs F1 BC with testosterone-exposed males, but it was increased in control females vs F1 BC-exposed males (5.34, 8.73, and 13.75% for 30, 60, and 90 mg BC/kg BW exposed, respectively). The mean number of resorptions per mice increased significantly (*P* < 0.05) in control females mated with F1 males exposed to BC. In contrast, no significant change was observed in the mean number of resorptions between control females vs control males and control females vs F1 BC with testosterone-exposed males. The percentage of the resorption index (6.67, 12.63, and 32.69%) and postimplantation loss (3.17, 6.12, and 9.36%) (Figure 2) increased in control females mated with F1 males exposed to BC, compared to control females vs control males. Comparable results were obtained between control females mated with control males and control females vs F1 BC with testosterone-exposed males in case of resorption index and postimplantation loss (Table 1 and Figure 2).

### 3.3. F1 Male Body Weights and Organ/Tissue Somatic Indices

**Figure 3:**
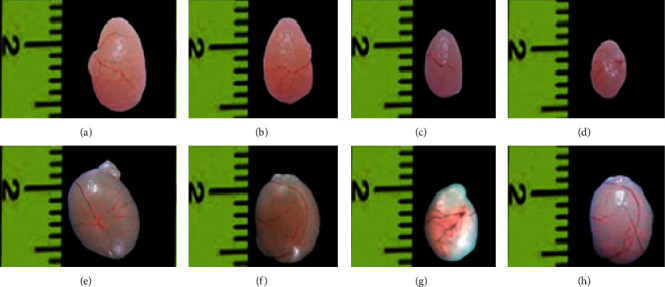
Size of testis in mice exposed to baicalein (BC) without or with testosterone (T) injection. (a) and (e) Control. (b), (c), and (d) 30, 60, and 90 mg·BC/kg body weight (BW) exposed F1 males, respectively; (f), (g), and (h) 30, 60, and 90 mg·BC/kg BW exposed F1 males administered with 4.16 mg·T/kg BW, respectively.

**Figure 4:**
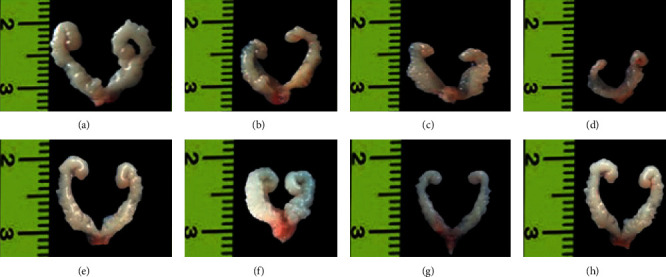
Seminal vesicle of mice exposed to prenatal baicalein (BC) without or with testosterone (T) injection. (a) and (e) Control. (b), (c), and (d) 30, 60, and 90 mg·BC/kg body weight (BW) exposed F1 males, respectively; (f), (g), and (h) 30, 60, and 90 mg BC/kg BW exposed F1 males administered with 4.16 mg T/kg BW, respectively.

The exposure of BC in F1 males at the age of 60 days significantly (*P* < 0.05; *F* = 7.11) decreased the mean body weights (±SEM) compared to control and F1 BC with testosterone-exposed males (Table 2). The BC-exposed F1 mice showed significant decrease in the relative weights of the liver (*P* < 0.001; *F* = 10.81), kidney (*P* < 0.001; *F* = 13.26), testis (*P* < 0.0001; *F* = 16.99) (Figure 3), epididymis (*P* < 0.05; *F* = 8.08), seminal vesicle (*P* < 0.0001; *F* = 52.99) (Figure 4), and prostate gland (*P* < 0.0001; *F* = 20.03) when compared to controls. Administration of testosterone to BC-exposed F1 males increased the relative organ weights such as kidney, (*P*=0.8642; *F* = 0.2431), testes (*P*=0.0877; *F* = 3) (Figure 3), epididymis (*P*=0.3945; *F* = 1.111) and seminal vesicle (*P*=0.0504; *F* = 3.848) (Figure 4) are comparable to that of controls. But the organ weights of liver (*P* < 0.05; *F* = 3.9) and prostate (*P* < 0.05; *F* = 5.931) did not significantly resume in BC-exposed F1 males with testosterone when compared to controls (Table 2). However, the mean weights of brain and spleen was comparable among control and BC-exposed with or without testosterone F1 males.

### 3.4. F1 Generation Spermatology

In BC-exposed F1 males observed significant decrease in percentage of motile sperm (*P* < 0.0001; *F* = 37.30), sperm count (*P* < 0.0001; *F* = 44.60), sperm viability (*P* < 0.0001; *F* = 28.35), HOS tail coiling (*P* < 0.001; *F* = 11.43) and daily sperm production (*P* < 0.0001; *F* = 20.22) in a dose dependent manner (Table 3). Administration of testosterone to F1 males exposed to BC resumed the sperm parameters motility (*P*=0.0685; *F* = 3.366), count (*P*=0.1319; *F* = 2.433), viability (*P*=0.059; *F* = 3.586) and HOS (*P*=0.8981; *F* = 0.1937), except DSP (*P*=0.035; *F* = 4.46) when compared to controls (Table 3).

### 3.5. F1 Male Steroidogenic Enzymes

**Figure 5:**
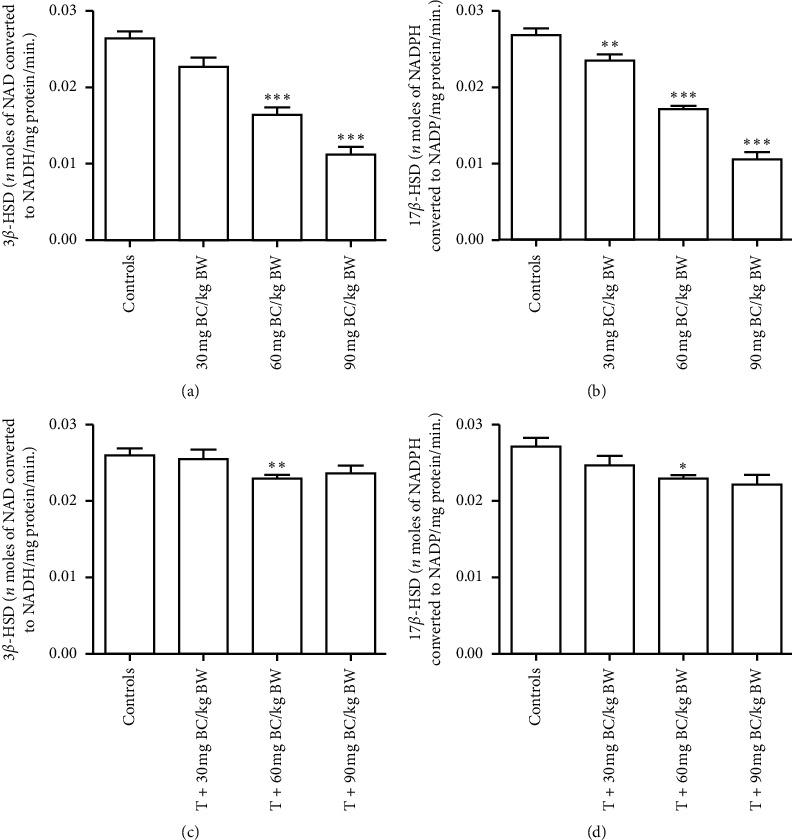
Testicular steroidogenic marker enzymes 3*β*-((a) and (c)) and 17*β*-((b) and (d)) hydroxy steroid dehydrogenase (3*β*-and 17*β*-HSD) titers in F1 adult mice exposed to baicalein (BC) without (*n* = 6) or with (*n* = 4) testosterone (T) injection. ^*∗*^, ^*∗∗*^, and ^*∗∗∗*^ represent significantly difference from controls at *P* < 0.05, <0.01, and < 0.001, respectively.

The activity levels of testicular 3*β*-HSD (*P* < 0.0001; *F* = 47.82) and 17*β*-HSD (*P* < 0.0001; *F* = 125.5) decreased significantly in F1 male mice exposed to BC as compared to controls (Figures 5(a) and 5(b)), whereas in F1 male mice subjected to BC exposure and testosterone supplementation, the testicular steroidogenic enzyme activity levels significantly increased as compared to their respective controls (3*β*-HSD: *P*=0.1091; *F* = 2.692 and 17*β*-HSD: *P*=0.0817; *F* = 3.103) (Figures 5(c) and 5(d)).

### 3.6. F1 Male Serum Reproductive Hormone Levels

Exposure to BC in F1 male mice significantly decreased (*P* < 0.0001; *F* = 38.64) serum testosterone levels in a dose-dependent manner as compared to controls (Table 4), whereas serum FSH (*P* < 0.0001; *F* = 109.5) and LH (*P* < 0.05; *F* = 6.529) levels increased significantly in BC-exposed F1 males over controls. Postnatal administration of testosterone to prenatally BC-exposed males resumed the testosterone (*P*=0.3197; *F* = 1.346) and LH (*P*=0.0133; *F* = 6.35) levels as compared to controls. However, partial resumption of serum FSH (*P* < 0.05; *F* = 0.887) levels was recorded in BC-exposed F1 males supplemented with testosterone over controls (Table 4).

### 3.7. Histopathology

**Figure 6:**
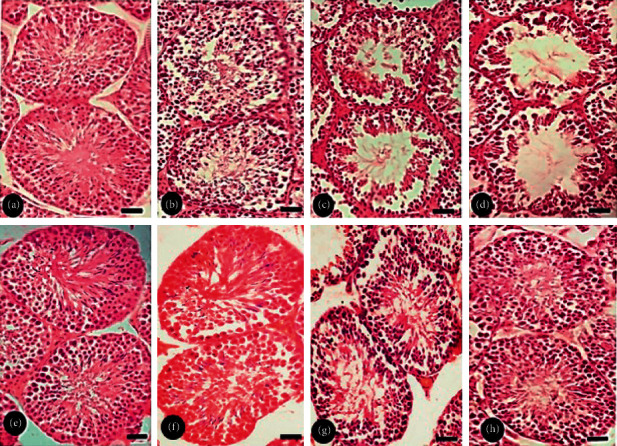
Transverse section of testes exposed to baicalein (BC) without or with testosterone (T) injection. Scale bar = 50 *μ*m. (a), (e) Testis of control mice showing the presence of normal tubular structure with spermatogenic cells at different stages of development. (b) Testis of F1 mice exposed to 30 mg·BC/kg body weight (BW) showing the wide lumen with a less number of sperm and spermatogenic cells. (c) Testis of F1 mice exposed to 60 mg·BC/kg BW showing wide intratubular space with a less number of sperm in the lumen. (d) Testis of F1 mice exposed to 90 mg·BC/kg BW showing large lumen devoid of sperm. (f), (g), and (h) Testis of F1 mice injected with 4.19 mg·T/kg BW to 30, 60, and 90 mg·BC/kg BW, respectively, showing seminiferous tubules filled with spermatozoa.

Histology sections of the control testes show well-developed seminiferous tubules with regular process of spermatogenesis and contain all cells. Clusters of Leydig cells are present in the interstitial spaces. Spermatogonia and Sertoli cells are present on the membrane of seminiferous tubule, and many spermatids are present in the middle of the lumen of tubule (Figures 6(a) and 6(e)). The histology of testes in 30 mg/kg BW BC-exposed F1 males shows seminiferous tubules with Leydig cells in interstitial spaces but the lumen with a less number of spermatids as compared to controls (Figure 6(b)). The testicular histopathology of F1 males exposed to 60 and 90 mg BC/kg BW exhibits adverse disruption testicular architecture as indicated by enlarged disordered seminiferous tubules with the lumen with a reduced number of spermatids (Figures 6(c) and 6(d)). On the other hand, the testicular organization was recovered in BC-exposed mice injected with testosterone as indicated by intact epithelium as basement membrane with lumen occupied by sperm (Figure 6).

## 4. Discussion

The differentiation of organ systems including the male reproductive tract during the critical window periods is more sensitive to chemical exposures than adults. Therefore, exposure of the male fetus to endocrine disrupting chemicals including phytoestrogens could have a negative impact on male reproductive health later in life [4]. The present study was designed to test this hypothesis. The results of the present study indicated that the mice exposed to baicalein at all selected doses (30, 60, and 90 mg/kg body weight) during prenatal period resulted in the following: (1) reduction in the body weight and weights of the liver, kidney, testis, and accessory sex organs, (2) reduced testicular functions, (3) altered sperm maturation events, (4) disrupted testicular architecture, (5) disturbances in the serum hormone levels of FSH, LH, and testosterone, and (6) reduced fertility efficacy.

In the present study, we found a significant reduction in the weights of body weights in BC-exposed mice over controls, suggesting that the overt metabolic condition of mice was deteriorated. This was followed by a significant reduction in the weights of the liver, kidney, testis, and accessory sex organs including epididymis, seminal vesicles, and prostate glands in BC-exposed mice suggesting that during the developmental window period, these organs could be susceptible to BC exposure. No significant changes were noticed in the weights of the spleen and brain in BC-exposed animals. Previously, significant reduction in the weights of reproductive organs was observed in experimental animals exposed to xenoestrogens [10, 35].

It is claimed that exposure of the male fetus to xenoestrogens during early life stages leads to reduced testis size and deterioration of sperm production in rodents [36]. The findings of this study indicated a significant reduction in the testicular daily sperm count and epididymal sperm variables including the sperm count, number of viable sperm, motile sperm, and HOS-coiled sperm associated in BC-exposed mice at all selected doses might support this notion. Our results also demonstrate that exposure to BC during the prenatal period not only interferes with Sertoli cell functions but also epididymal sperm maturation events. The results are in agreement with previous studies [8, 10]. Significant reduction in the viable sperm and motile sperm was reported in xenoestrogen- and genistein-exposed rats and mice, respectively [8, 9]. Deterioration of spermatogenesis via loss of germ cells has been reported in adult male rats exposed to phytoestrogens [37]. In another study, it has been indicated that exposure of mice to phytoestrogens from conception to adulthood showed reduction in epididymal sperm count [35]. Excess consumption of dietary phytoestrogens alters the process of spermatogenesis, which leads to male infertility [38]. The dietary isoflavonoid exposure disrupts the hypothalamic-pituitary-gonadal axis and results in low sperm count and quality in animals (https://www.sciencedirect.com/science/article/pii/S0160412019303496) [39].

Testicular spermatogenesis and epididymal sperm maturation events largely depend on adequate supply of androgens. It is a well-known fact that the bioavailability of androgens is considered important for the structural and functional integrity of the testis and accessory sex organs [40]. The biosynthesis of testosterone occurs in the testicular Leydig cells wherein 3*β*- and 17*β*-HSDs play a key role in the conversion of cholesterol to testosterone. In the present study, prenatal exposure to BC significantly reduced the activity levels of testicular 3*β*- and 17*β*-HSDs in F1 mice, suggesting reduced testicular steroidogenesis. Accordingly, we found a significant reduction in the circulatory testosterone levels in experimental mice [10, 41]. In addition to the testosterone, the gonadotropins such as FSH and LH also play a key role in Sertoli cell spermatogenesis and Leydig cell steroidogenesis, respectively. Significant decrease in the serum testosterone levels accompanied by significant increase in the serum FSH and LH levels in BC-exposed mice at higher dose could reflect the compromised hypothalamo-pituitary-testicular axis [42]. This might also reflect the direct effect of BC at the level of Leydig cell steroidogenesis and/or indirectly due to the lack of Leydig cell responsiveness against the LH [10]. Previously, it has been shown that the exposure of rats to estrogens or its mimics during the neonatal period negatively affect the steroidogenic capacity of Leydig cells at their adulthood [43]. Earlier, it was reported that the exposure of rats to weak estrogenic compounds can enhance FSH levels with decreased plasma testosterone levels [44]. We also found that mice exposed to BC at all selected doses (30, 60, or 90 mg/kg body weight) enhanced the serum FSH levels but enhancement in the serum LH levels was observed only in mice exposed to BC at 90 mg/kg body weight. Studies of Svechnikov et al. [45] showed that the dietary exposure to genistein did not affect serum LH and testosterone levels but reduced Leydig cell steroidogenesis. Studies also indicated that mice fed dietary genistein *in utero* resulted in no significant changes in the serum LH and androgen levels associated with a significant reduction in the transcripts for androgen response genes in the Sertoli cells and a germ cell specific gene, *Gapd-s*, associated with sperm glycolysis and mobility [35]. From the above, it is evident that the diminished spermatogenesis and sperm maturation events in experimental mice could be primarily due to inadequate supply of androgens. Moreover, reduction in the serum testosterone levels with concomitant increase in the serum FSH and LH in experimental mice could reflect improper testosterone-mediated feedback mechanisms at the level of testicular-pituitary axis. Previously, it has been shown that male rats treated with dietary 17*β*-estradiol showed reduced serum testosterone levels [46]. The degenerative changes observed in the structural integrity of testicular architecture in BC-exposed mice could be attributed to the inadequate supply of androgen levels [10].

It is well known that the fertility efficacy of an individual is determined by normal testicular steroidogenesis, spermatogenesis, and epididymal sperm maturation events. Thus, alterations at this level affect fertility ability of individual. Thus, fertility assays are considered as one of potential reproductive endpoints to evaluate the reproductive performance of an individual to sire an offspring in a fixed point of time. In the present study, increase in the preimplantation loss with a reduced number of live pups in females cohabited with mice exposed to BC at 60 or 90 mg/kg bodyweight might suggest reduced reproductive performance. The results are in agreement with the previous studies [8, 10]. It is well established that the sperm with reduced motility cannot reach the zona pellucida of ova, consequently impairment in fertilization capacity of sperm. Thus, a reduction in the sperm motility and its integrity (the HOS-test) associated with suppressed male reproductive performance in mice exposed to BC may reflect compromised sperm fertility.

Testosterone is widely prescribed to patients diagnosed with infertility problems [47]. Furthermore, the supplementation of testosterone to combat male reproductive ailments against the pharmaceutical drugs and cadmium is well acknowledged [23, 48]. The results obtained from the present study also support the above notion where testosterone administration during postnatal days 21, 31, and 41 alleviates the baicalein-induced reproductive toxic effects in F1 mice as evidenced by enhanced reproductive organ weights, increased sperm quality and quantity and improvement in the activity levels of testicular steroidogenic marker enzymes and testicular architecture and restoration of fertility outcome. The exact mechanism of action of testosterone-mediated amelioration of suppressed male reproduction in F1 mice exposed to BC prenatally cannot be determined from this study. However, it is well known that testosterone promotes testicular growth and supports the Sertoli cell functions [49]. Thus, reparative effects of testosterone on sperm count and sperm maturation events associated with enhanced testicular and accessory sex organ weights in mice exposed to baicalein might be attributed to the adequate supply of testosterone. The other plausible reason could be that the exogenous supply of testosterone could sustain that the physiological levels of testosterone thereby support the testicular and epididymal functions [23, 42]. Furthermore, restoration of testicular architecture in testosterone-treated BC-exposed mice could plausibly ameliorate testicular steroidogenesis [48]. The regulation of FSH and LH levels are dependent on the feedback system operated at the level of pituitary-testicular axis, testosterone being the central candidate. In the present study, partial recovery in the serum FSH and LH levels was observed in F1 mice exposed to BC administered with testosterone as compared to its respective controls, suggesting that the testosterone supplemented exogenously might interfere with the feedback regulation of gonadotropins. The sperm endpoints such as testicular daily sperm count, epididymal sperm variables including sperm motility, and sperm membrane integrity were enhanced in testosterone-injected BC-exposed mice. These events were consequently responsible for the enhanced fertility efficacy in testosterone-treated male mice. To summarize, our results indicated that male reproductive health was recovered by postnatal administration of testosterone in F1 mice exposed to BC prenatally. Previously, it has been shown that administration of testosterone showed restoration of testicular and accessory organ weights, enhancement of sperm count and sperm motility, increased activity levels of testicular steroidogenic enzymes, and reduced levels of LH and FSH levels with a concomitant increase in testosterone levels associated with fertility ability of mice exposed to progesterone transplacentally [23].

## 5. Conclusions

In conclusion, we have shown that the testosterone treatment repairs reproductive ailments caused by baicalein in male mice exposed during the prenatal period. The present study also cautions that the consumption of baicalein by pregnant women might be harmful as the offspring male mice developed deteriorated reproductive health later in life. Furthermore, the information obtained from this study will be useful to develop therapeutic strategies to negate some of the male fertility problems.

## Figures and Tables

**Table 1:** Administration of testosterone (T) on reproductive performance of adult male mice exposed to baicalein (BC) prenatally.

Parameters	Group	Control/BC-exposed groups	4.16 mg· T/kg BW (BC + T) (#)
Mating index (%)	Control	100 (6/6)	100 (6/6)
	30 mg	100 (6/6)	100 (6/6)
	60 mg	100 (6/6)	100 (6/6)
	90 mg	100 (6/6)	100 (6/6)
Fertility index (%)	Control	100%	100%
	30 mg	83.3%	100%
	60 mg	66.6%	75%
	90 mg	50%	75%
No. of corpora lutea/mice	Control	13.5 ± 0.22	13.25 ± 0.25
	30 mg	12.66 ± 0.21 (−6.22), *P*=0.004	12.00 ± 0.0 (−7.54), *P*=0.0154
	60 mg	11.83 ± 0.40 (−12.37), *P*=0.019	13.00 ± 0.0 (−1.88), *P*=0.3910
	90 mg	12 ± 0.44 (−11.11), *P*=0.017	12.50 ± 0.28 (−5.66), *P*=0.017
		*P*=0.0126, *F* = 5.082	*P*=0.1482, *F* = 2.280
Implantation (%)	Control	100	100
	30 mg	95.94	100
	60 mg	91.26	95.80
	90 mg	86.24	98.07
No. of implantations/mice	Control	11.16 ± 0.31	10.75 ± 0.25
	30 mg	10.5 ± 0.22 (−5.97), *P*=0.025	10.25 ± 0.29 (−2.32), *P*=0.3910
	60 mg	8.16 ± 0.31 (−26.86), *P* < 0.0001	10.25 ± 0.25 (−4.65), *P*=0.3910
	90 mg	5.3 ± 0.55 (−52.23), *P*=0.0001	10.25 ± 0.47 (−5.65), *P*=0.1817
		*P* < 0.0001, *F* = 92.36	*P*=0.6867, *F* = 0.5077
No. of live fetus/mice	Control	10.83 ± 0.166	10.00 ± 0.41
	30 mg	10.17 ± 0.16 (−6.15), *P*=0.025	9.50 ± 0.29 (−5), *P*=0.3910
	60 mg	7.67 ± 0.42 (−29.23), *P*=0.0012	9.750 ± 0.25 (−2.5), *P*=0.3910
	90 mg	4.83 ± 0.48 (−55.38), *P* < 0.0001	10.00 ± 0.41 (0), *P*=1.000
		*P* < 0.0001, *F* = 89.69	*P*=0.7761, *F* = 0.3708
Resorption (%)	Control	0	0
	30 mg	5.34	0
	60 mg	8.73	1.92
	90 mg	13.75	1.92
No. of resorptions/mice	Control	0	0
	30 mg	0.67 ± 0.42, *P*=0.174	0, *P*=1.000
	60 mg	1.00 ± 0.36, *P*=0.0409	0.5 ± 0.28, *P*=0.1817
	90 mg	1.83 ± 0.47, *P*=0.0121	0.25 ± 0.25, *P*=0.3910
		*P*=0.026, *F* = 4.088	*P*=0.1936, *F* = 1.941
Resorption index (%)	Control	0	0
	30 mg	6.67	0
	60 mg	12.63	2.5
	90 mg	32.69	2.27
Postimplantation loss (%)	Control	2.98	0
	30 mg	3.17	2.38
	60 mg	6.12	2.44
	90 mg	9.36	4.87

Values are mean ± SEM of six animals. # denotes *n* = 4. Values in the parentheses are percent changes from that of control (“−” indicates decrease). Significance was checked from controls to BC exposed and prenatal BC exposed with T groups. Significance was considered at *P* < 0.05, where BW = body weight.

**Table 2:** Administration of testosterone (T) on body weight (gm) and tissue indices (W/W%) of adult male mice exposed to baicalein (BC) prenatally.

Tissue	Group	Control/BC-exposed groups	4.16 mg T/kg·BW (BC + T) (#)
Body weight	Control	38.02 ± 0.91	38.87 ± 1.45
	30 mg	35.71 ± 0.84 (6.07), *P*=0.0345	35.22 ± 0.41(−9.39),*P*=0.119
	60 mg	35.35 ± 0.74 (−7.02), *P*=0.0016	35.45 ± 0.71 (−8.78), *P*=0.1813
	90 mg	35.36 ± 0.56 (−7.00), *P*=0.0062	36.84 ± 0.96 (−5.22), *P*=0.3606
		*P*=0.0034, *F* = 7.111	*P*=0.1079, *F* = 2.706
Brain	Control	0.46 ± 0.01	0.45 ± 0.0008
	30 mg	0.40 ± 0.02 (−13.48), *P*=0.0521	0.43 ± 0.01 (−4.03), *P*=0.086
	60 mg	0.42 ± 0.02 (−9.03), *P*=0.1296	0.43 ± 0.01 (−4.14), *P*=0.269
	90 mg	0.35 ± 0.03 (−22.94), *P*=0.0395	0.44 ± 0.02 (−2.26), *P*=0.599
		*P*=0.1117, *F* = 2.368	*P*=0.6440, *F* = 0.5778
Liver	Control	2.59 ± 0.11	2.52 ± 0.068
	30 mg	1.95 ± 0.16 (−24.91), *P*=0.0359	2.25 ± 0.03 (−10.75), *P*=0.0336
	60 mg	1.69 ± 0.15 (−34.78), *P*=0.0006	2.29 ± 0.11 (−8.91), *P*=0.0916
	90 mg	1.58 ± 0.13 (−0.15), *P*=0.0021	2.27 ± 0.10 (−9.91), *P*=0.0270
		*P*=0.0005, *F* = 10.81	*P*=0.0489, *F* = 3.900
Kidney	Control	0.84 ± 0.08	0.83 ± 0.118
	30 mg	0.74 ± 0.07 (−11.77), *P*=0.0089	0.81 ± 0.01 (−2.27), *P*=0.889
	60 mg	0.49 ± 0.07 (−41.87), *P*=0.0186	0.80 ± 0.02 (−3.29), *P*=0.844
	90 mg	0.42 ± 0.03 (−50.13), *p*=0.0042	0.76 ± 0.01 (−8.68), *P*=0.564
		*P*=0.0002, *F* = 13.26	*P*=0.8642, *F* = 0.2431
Spleen	Control	0.22 ± 0.012	0.22 ± 0.015
	30 mg	0.17 ± 0.01 (−25.49), *P*=0.0021	0.21 ± 0.001 (−5.22), *P*=0.536
	60 mg	0.19 ± 0.05 (−13.65), *P*=0.6305	0.19 ± 0.02 (−16.21), *P*=0.108
	90 mg	0.14 ± 0.02 (−34.33), *P*=0.0142	0.21 ± 0.02 (−8.59), *P*=0.612
		*P*=0.3860, *F* = 1.084	*P*=0.2035, *F* = 1.879
Testis	Control	0.275 ± 0.011	0.26 ± 0.013
	30 mg	0.21 ± 0.02 (23.29), *P*=0.0577	0.23 ± 0.01 (−11.42), *P*=0.259
	60 mg	0.19 ± 0.01 (−29.46), *P*=0.0030	0.21 ± 0.01 (−22.09), *P*=0.099
	90 mg	0.15 ± 0.01 (−46.64), *P* < 0.0001	0.23 ± 0.01 (−14.13), *P*=0.031
		*P* < 0.0001, *F* = 16.99	*P*=0.0877, *F* = 3.000
Epididymis	Control	0.90 ± 0.058	0.99 ± 0.070
	30 mg	0.84 ± 0.03 (−11.14), *P*=0.2009	0.90 ± 0.01 (−8.38), *P*=0.5691
	60 mg	0.74 ± 0.05 (−27.20), *P*=0.0288	0.89 ± 0.03 (−9.61), *P*=0.352
	90 mg	0.67 ± 0.06 (−28.58), *P*=0.0033	0.92 ± 0.03 (−7.09), *P*=0.285
		*P*=0.0019, *F* = 8.08	*P*=0.3945, *F* = 1.111
Seminal vesicle	Control	0.26 ± 0.0028	0.26 ± 0.001
	30 mg	0.22 ± 0.003 (−15.10), *P*=0.0003	0.26 ± 0.001, (−1.04), *P*=0.184
	60 mg	0.18 ± 0.001 (−29.45), *P*=0.0029	0.25 ± 0.003 (−4.65), *P*=0.083
	90 mg	0.11 ± 0.004 (−95.90), *P* < 0.0001	0.26 ± 0.002 (−2.27), *P*=0.0115
		*P* < 0.0001, *F* = 52.99	*P*=0.0504, *F* = 3.848
Prostate	Control	0.07 ± 0.003	0.08 ± 0.004
	30 mg	0.06 ± 0.004 (−13.13), *P*=0.0330	0.06 ± 0.001, (−7.06), *P*=0.0521
	60 mg	0.05 ± 0.004 (−27.07), *P*=0.0062	0.06 ± 0.001 (−13.19), *P*=0.018
	90 mg	0.04 ± 0.01 (−40), *P*=0.0023	0.05 ± 0.001 (−5.27), *P*=0.008
		*P* < 0.0001, *F* = 20.03	*P*=0.0162, *F* = 5.931

Values are mean ± SEM of six animals. # denotes *n* = 4. Values in the parentheses are percent changes from that of control (“−” indicates decrease). Significance was checked from control males to F1 BC exposed and BC exposed administered with T groups. Significance was considered at *P* < 0.05, where BW = body weight.

**Table 3:** Administration of testosterone (T) on sperm parameters of adult male Wistar mice exposed to baicalein (BC) prenatally.

Parameters	Group	Control/BC-exposed groups	4.16 mg T/kg·BW (BC + T) (#)
Sperm motility (%)	Control	67.67 ± 2.27	68.00 ± 2.944
	30 mg	55.17 ± 1.93 (−18.47), *P*=0.0034	63.5 ± 1.19 (−6.61), *P*=0.275
	60 mg	44.50 ± 3.44 (−34.23), *P*=0.0041	61.25 ± 1.49 (−9.93), *P*=0.195
	90 mg	31.67 ± 0.84 (−53.20), *P* < 0.0001	60.25 ± 0.85 (−11.39), *P*=0.058
		*P* < 0.0001, *F* = 37.30	*P*=0.0685, *F* = 3.366
Sperm count (millions/ml)	Control	69.83 ± 2.07	70.00 ± 1.958
	30 mg	63.17 ± 2.93 (−9.54), *P*=0.0237	66.75 ± 0.75 (−4.64), *P*=0.135
	60 mg	54.00 ± 1.12 (−22.67), *P*=0.0023	66.25 ± 1.31 (−5.37), *P*=0.257
	90 mg	45.67 ± 1.11 (−34.60), *P* < 0.0001	64.25 ± 1.79 (−8.94), *P*=0.133
		*P* < 0.0001, *F* = 44.60	*P*=0.1319, *F* = 2.433
Viability (%)	Control	70.33 ± 1.40	72.00 ± 1.225
	30 mg	66.50 ± 2.77 (−5.45), *P*=0.0692	69.50 ± 0.87 (−3.47), *P*=0.287
	60 mg	56.50 ± 1.54 (−19.66), *P*=0.0013	67.50 ± 0.96 (−2.27), *P*=0.093
	90 mg	48.17 ± 1.92 (−31.51), *P*=0.0004	68.25 ± 0.63 (−5.28), *P*=0.0094
		*P* < 0.0001, *F* = 28.35	*P*=0.059, *F* = 3.586
HOS (%)	Control	61.00 ± 1.82	59.25 ± 2.287
	30 mg	54.12 ± 1.51 (−11.20), *P*=0.0645	58.25 ± 0.47(−1.68), *P*=0.689
	60 mg	48.00 ± 3.35 (−21.31), *P*=0.0156	57.75 ± 2.83(−2.53), *P*=0.695
	90 mg	43.00 ± 1.39 (−29.51), *P*=0.0457	57 ± 1.47(−3.79), *P*=0.590
		*P*=0.0004, *F* = 11.43	*P*=0.8981, *F* = 0.1937
DSP (millions/gram testis)	Control	23.33 ± 0.61	23.50 ± 0.86
	30 mg	19.33 ± 1.08 (−17.14), *P*=0.0250	21.5 ± 0.28 (−8.51), *P*=0.066
	60 mg	15.50 ± 0.95(−33.57), *P*=0.0008	20.25 ± 0.63 (−13.83), *P*=0.051
	90 mg	12.50 ± 1.25(−46.42), *P* < 0.0001	22 ± 1.00 (−6.38), *P*=0.181
		*P* < 0.0001, *F* = 20.22	*P*=0.0350, *F* = 4.468

Values are mean ± SEM of six individuals; # denotes *n* = 4. Values in the parentheses are percent changes from that of control. Significance was checked from controls to F1 BC exposed and BC exposed administered with T groups. Significance was considered at *P* < 0.05, where BW = body weight; HOS = hypoosmotic solution; and DSP: daily sperm count.

**Table 4:** Administration of testosterone on the levels of serum testosterone (T), follicle stimulating hormone (FSH), and luteinizing hormone (LH) of adult male Wistar mice exposed to baicalein (BC) prenatally.

Hormone	Group	Control/BC-exposed groups	4.16 mg T/kg·BW (BC + T) (#)
Testosterone (ng/ml)	Control	7.38 ± 0.11	7.39 ± 0.15
	30 mg	7.14 ± 0.13 (−3.34), *P*=0.1307	7.34 ± 0.13 (−0.68), *P*=0.6297
	60 mg	6.54 ± 0.13 (−11.48), *P*=0.0084	7.25 ± 0.12 (−1.93), *P*=0.5950
	90 mg	5.61 ± 0.11 (−23.94), *P*=0.0002	7.02 ± 0.16 (−5.04), *P*=0.2764
		*P* < 0.0001, *F* = 38.64	*P*=0.3197, *F* = 1.346
FSH (ng/ml)	Control	4.55 ± 0.11	4.57 ± 0.18
	30 mg	5.36 ± 0.15 (17.99), *P*=0.0121	4.71 ± 0.18 (3.17), *P*=0.6142
	60 mg	8.03 ± 0.18 (76.75), *P* < 0.0001	5.54 ± 0.18 (21.33), *P*=0.0422
	90 mg	9.84 ± 0.33 (116.49), *P* < 0.0001	5.87 ± 0.29 (28.39), *P*=0.0124
		*P* < 0.0001, *F* = 109.5	*P*=0.0060, *F* = 0.8870
LH (ng/ml)	Control	1.59 ± 0.15	1.57 ± 0.158
	30 mg	1.62 ± 0.231 (1.98), *P*=0.9151	1.60 ± 0.22 (1.42), *P*=0.862
	60 mg	2.12 ± 0.27 (33.40), *P*=0.1374	1.88 ± 0.25 (19.17), *P*=0.3271
	90 mg	2.86 ± 0.28 (79.79), *P*=0.0091	2.23 ± 0.26 (45.64), *P*=0.0498
		*P*=0.0048; *F* = 6.529	*P*=0.0133; *F* = 6.35

Values are mean ± SEM of six animals; # denotes *n* = 4. Values in the parentheses are percent changes from that of control. Significance was checked from control males to F1 BC exposed and BC exposed administered with T groups. Significance was considered at *P* < 0.05.

## Data Availability

The statistical data of the present study are available from the corresponding author upon reasonable request.
